# Correlation of alanine aminotransferase levels and a histological diagnosis of steatohepatitis with ultrasound-diagnosed metabolic-associated fatty liver disease in patients from a centre in Nigeria

**DOI:** 10.1186/s12876-024-03237-4

**Published:** 2024-05-09

**Authors:** O. J. Kolawole, M. M. Oje, O. A. Betiku, O. Ijarotimi, O. Adekanle, D. A. Ndububa

**Affiliations:** 1 Department of General Medicine, Frimley Health NHS Foundation Trust, Wexham Park Hospital, Slough, SL2 4HL UK; 2 Department of Medicine, LAUTECH Teaching Hospital, Ogbomoso, Oyo State Nigeria; 3 Department of Morbid Anatomy, College of Health Sciences, Obafemi Awolowo University Teaching Hospital Complex, Ile-Ife, Osun State Nigeria; 4 Department of Medicine, Faculty of Clinical Sciences, Obafemi Awolowo University and Obafemi Awolowo University Teaching Hospital Complex, Ile-Ife, Osun State Nigeria

**Keywords:** MAFLD, NASH, Liver biopsy, Nigeria, Alanine aminotransferase

## Abstract

**Background:**

Metabolic-associated fatty liver disease (MAFLD) is defined as the occurrence of hepatic fat accumulation in patients with negligible alcohol consumption or any other cause of hepatic steatosis. This study aimed to correlate the ultrasound-based diagnosis of MAFLD with the histological diagnosis of nonalcoholic steatohepatitis (NASH) and alanine aminotransferase (ALT) levels in patients with MAFLD.

**Methods:**

This was a hospital-based cross-sectional study of 71 patients with MAFLD diagnosed by ultrasound. Percutaneous liver biopsy was performed for histological evidence of NASH in all patients, regardless of liver function test (LFT) values, provided that they had no contraindications. Liver histology was graded using the NASH Clinical Research Network MAFLD Activity Score. The data obtained were entered into SPSS version 21 and analysed using descriptive and inferential statistics. The significance level was set at < 0.05.

**Results:**

A total of 71 patients (26 males and 45 females) with MAFLD were included. Thirty-nine (76.5%) patients with MAFLD and normal ALT levels had NASH, while 14 (82.4%) had elevated ALT levels. There was no statistically significant difference in the histological grade of NASH between patients with normal and elevated ALT levels. A weak correlation was found between the severity of steatosis on ultrasound scan and NASH incidence (*p* = 0.026). The sensitivity and specificity of ALT levels for predicting NASH according to the area under the receiver operating characteristics (AUROC 0.590) at an ALT cut-off value of 27.5 IU/L were 55.8% and 64.7%, respectively.

**Conclusion:**

NASH can occur in patients with MAFLD, irrespective of alanine transaminase (ALT) levels, and ultrasound grading of the severity of steatosis cannot accurately predict NASH. Liver biopsy remains the investigation of choice.

## Introduction

Metabolic-associated fatty liver disease (MAFLD) is defined as the occurrence of hepatic fat accumulation after the exclusion of other causes of hepatic steatosis, such as liver disease and excessive alcohol consumption [1]. In clinical practice, MAFLD is regarded as the presence of fatty liver on ultrasonography in the absence of known secondary causes of fatty liver disease [2]. Nonalcoholic steatohepatitis (NASH) is a histologic category of MAFLD characterized by hepatic steatosis and inflammation with hepatocyte injury with or without fibrosis [3]. NASH can progress to cirrhosis, liver failure and hepatocellular carcinoma and is the third most common risk factor for hepatocellular carcinoma after viral hepatitis and alcohol consumption [2]. MAFLD is one of the most common liver diseases worldwide, and NASH may soon become the most common indication for liver transplantation [3]. MAFLD affects approximately 80 to 100 million individuals in the USA, among whom nearly 25% progress to NASH [1].

Liver biopsy is the gold standard for the diagnosis of MAFLD because it is the only way of assessing the presence of inflammation and fibrosis [4].

A study in Nigeria reported the prevalence of fatty liver to be 8.7% [5] and that of MAFLD to be 1.38% [6]. The increasing incidence of MAFLD because of increasing rates of obesity, type 2 DM and dyslipidaemia is worrisome. NASH is the most important component of the MAFLD spectrum, irrespective of the aminotransferase level. There is a paucity of literature on the indications for liver biopsy in patients with MAFLD with respect to their serum alanine aminotransferase (ALT) level to perform NASH assessment and provide prompt intervention. According to a population study in Sweden [7], once MAFLD progresses to NASH, cirrhosis survival decreases. The objectives of this study were therefore to determine the proportion of patients with MAFLD and normal ALT levels who have histological evidence of NASH and the proportion of patients with MAFLD and elevated ALT levels who have NASH; to assess the MAFLD activity score in patients with MAFLD and NASH; and to relate the histological findings to the ultrasound grading of fatty liver disease. Hence, there is a need for this study in Nigeria.

## Materials and methods

This was a hospital-based cross-sectional study of 71 patients with MAFLD. The study period was from July 2019 to July 2021. The study was carried out in the gastroenterology clinic of a university teaching hospital in southwest Nigeria. The study included patients who were aged 18 years and older and had an ultrasound diagnosis of fatty liver disease and were either visiting the gastroenterology clinic or referred from the general outpatient, endocrinology and metabolic clinic or the cardiology clinic of the teaching hospital who met the inclusion criteria.

The inclusion criteria were as follows:

1. Individuals aged 18 years and older with an ultrasound diagnosis of fatty liver who were diagnosed by a specialist radiologist using the conventional USG system and graded according to fatty liver disease severity (grade 1: when liver echogenicity is just increased; grade 2: when the echogenic liver obscures the echogenic walls of the portal vein branches; and grade 3: when the echogenic liver obscures the diaphragmatic outline).
2. Patients with fatty liver disease and no significant alcohol consumption (≤ 21 units/week or < 30 g/day for men and ≤ 14 units/week or < 20 g/day for women) [1, 8].
  **Hypertension** in the study subjects was defined as a blood pressure ≥ 140/90 mmHg, which was checked one time, or on the use of antihypertensive drugs.
  **Diabetes status** was defined according to the World Health Organization (WHO) guidelines [9] as follows:
  - A fasting plasma glucose level greater than or equal to 7 mmol/L (126 mg/dl).
    AND/OR
  - A 2-hour postprandial plasma glucose level greater than or equal to 11.1 mmol/L (200 mg/dl).
  - Already receiving treatment for DM.

**Obesity** was defined as a body mass index ≥ 30 kg/m^2^, a waist circumference > 102 cm (40 inches) in men and > 88 cm (35 inches) in women and/or a waist/hip ratio > 0.90 in men and > 0.85 in women [10].

**Dyslipidaemia** was defined as having one or more of the following: an LDL-C level ≥ 100 mg/dl (2.6 mmol/l), a total cholesterol (TC) level ≥ 200 mg/dl (5.18 mmol/l), a triglyceride level ≥ 150 mg/dl (1.7 mmol/l) or a high-density lipoprotein cholesterol (HDL) level ≤ 40 mg/dl (1 mmol/l) in men and ≤ 50 mg/dl (1.3 mmol/l) in women [11].

Patients with the following conditions were excluded:

1. Patients with hepatitis B virus, hepatitis C virus or HIV infection.
2. Patients with a history of medication use, such as glucocorticoid and synthetic oestrogen, amiodarone, tamoxifen, methotrexate, tetracycline, antiretroviral drugs, or toxic mushrooms.
3. Patients with comorbidities such as congestive heart failure, chronic obstructive pulmonary disease, end-stage chronic kidney disease and malignancy of any origin.
4. Pregnant women.

The sample size was calculated using the formula for estimating single proportions and a prevalence of MAFLD (8.7%) [5] as follows: ${\rm{n = }}\frac{{{\rm{Z}}{{\rm{\alpha }}^{\rm{2}}}{\rm{P}}{{\rm{q}}^{{\rm{12}}}}}}{{{{\rm{d}}^{\rm{2}}}}}$ [12]. This resulted in a sample size of 71. Eligible patients were recruited consecutively until the sample size was met.

A structured proforma was used to obtain the following: sociodemographic information; data on the history of the present illness, alcohol intake, psychoactive substance use and drug use; risk factors for MAFLD (hypertension, type 2 diabetes mellitus, and obesity) and liver disease; and physical examination, laboratory investigation, ultrasonography, liver biopsy and final diagnosis data.

All patients who consented to participate in the study had basic laboratory test results, which included full blood count, viral hepatitis B and C, and clotting profile data in addition to liver function test data obtained using an agape kit and processed using an automated Cobas machine via a method comparable to the International Federation of Clinical Chemistry guide. All patients underwent percutaneous liver biopsy irrespective of liver function test results. Liver biopsies were prepared according to standard guidelines, stained with appropriate stains and read by a pathologist with a special interest in liver histology. Liver histological evidence of steatohepatitis (NASH) included steatosis of more than 5% hepatocytes, inflammatory cell infiltrates, ballooning degeneration with or without Mallory bodies, and peri-cellular/peri-venular fibrosis [1]. NASH was classified using the NASH CRN score (steatosis + ballooning + lobular inflammation). Patients with a NASH score of 5 and above were considered to have definite NASH, while those with a score of 1–4 were classified as not having NASH [13].

Ethical approval was obtained from the hospital Ethics and Research Committee under IRB/IEC/0004553 and NHREC/27/02/2009a and protocol number ERC/2019/06/12. Informed and written consent was obtained from all the participants after providing a detailed explanation of the purpose of the study, procedures, and liver biopsy. The data obtained were entered into the Statistical Package for Social Sciences version (SPSS) version 21. The data were analysed using descriptive and inferential statistics. The significance level was set at a *p* value < 0.05 and a 95% confidence interval.

## Results

A total of 71 patients were recruited and completed the study. The age range was 19 years to 69 years, with a mean age (SD) of 50.99 (± 11.76) years. There were more females than males (63.4% vs. 36.6%). The majority of the participants were of Yoruba ethnicity (93%), were married (90.14%) and practised Christianity (87.3%). More than half (44; 62%) of the participants had a tertiary education, and most of them were either civil servants (33; 46.5%) or traders (24; 33.8%) (Table 1).

**Table 1:** Sociodemographic characteristics of the study participants

Variables	*N* % *n* = 71
**Age (mean ± SD)**	50.99 (± 11.76)
**Sex**	
Male Female	26 (36.6) 45 (63.4)
**Marital Status**	
Married Single Widowed	64 (90.14) 4 (5.63) 3 (4.23)
**Level of education**	
Primary Secondary Tertiary	1 (1.4) 26 (36.6) 44 (62.0)
**Ethnicity**	
Yoruba Igbo *Other	66 (93.0) 3 (4.2) 2 (2.8)
**Religion**	
Islam Christianity	9 (12.7) 62 (87.3)
**Occupation**	
Civil servant Trader Artisan Unemployed	33 (46.5) 24 (33.8) 5 (7.0) 9 (12.6)

Approximately 37 (52.1%) of the participants had symptoms, and among them,36 (50.7%) had right upper abdominal pain. The majority of the participants had hepatomegaly (51 [71.8%] patients) on abdominal examination. Only 11 (15.5%) patients were taking statins (Table 2).

**Table 2:** Symptoms and clinical findings of the study patients

Variables	*N* (%)
**Upper abdominal pain**	
Yes No	36 (50.7) 35 (49.3)
**Early satiety**	
Yes No	1 (1.4) 70 (98.6)
**Weight loss**	
Yes No	3 (4.2) 68 (95.8)
**Hepatomegaly**	
Yes No	51 (71.8) 20 (28.2)
**Drug history (statins)**	
Yes No	11 (15.5) 60 (84.5)

The medical conditions of the patients were as follows: hypertension in 43 patients (60.6%), dyslipidaemia in 52 patients (73.2%) and type 2 DM or impaired fasting in 13 patients (18.3%). Twenty (76.9%) of the males and 42 (93.3%) of the females had central obesity. According to BMI, 37 (52.1%) of the participants were obese, of whom 3 (4.2%) and 28 (39.4%) were morbidly obese and overweight, respectively (Table 3).

**Table 3:** Identified risk factors for MAFLD among the study participants

Variables	*N* (%) *N* = 71
**Hypertension**	
Yes No	43 (60.6) 28 (39.4)
**Type 2 DM**	
Yes No	13(18.3) 58 (81.7)
**Dyslipidaemia**	
Yes No	52 (73.2) 19 (26.8)
**Elevated 2HrPP**	
Yes No	13 (18.3) 58 (81.7)
**Impaired fasting glucose**	
Yes No	13 (18.3) 58 (81.7)
**BMI**	
25-29.9 (Overweight) 30-34.9 (Mild obesity) 35-39.9 (Moderate obesity) > 40 (Severe obesity)	28 (39.4) 22 (31.0) 15 (21.1) 3 (4.2)
**Central obesity**	
**Females**	
Waist-hip ratio > 0.85	42 (93.3)
**Males**	
Waist-hip ratio > 0.90	20 (76.9)

**Fig. 1:**
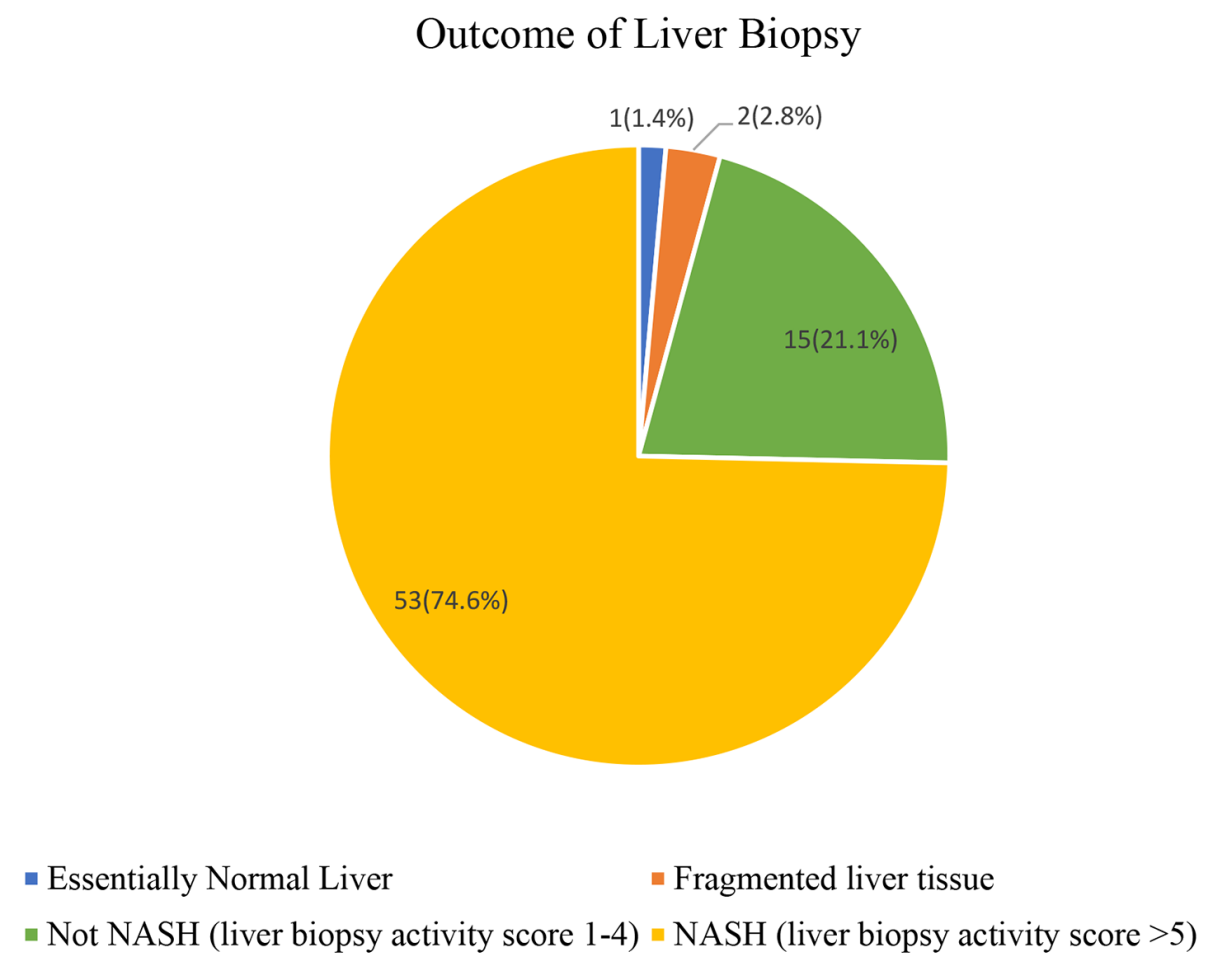
Outcome of liver biopsy

Histology of the 71 liver biopsy samples revealed that 1 (1.4%) participant had a normal liver, 2 (2.8%) participants had fragmented liver tissue that could not be characterized using the NASH CRN score, 53 (74.6%) participants had NASH, and 15 (21.1%) participants did not have NASH (Fig. 1).

**Fig. 2:**
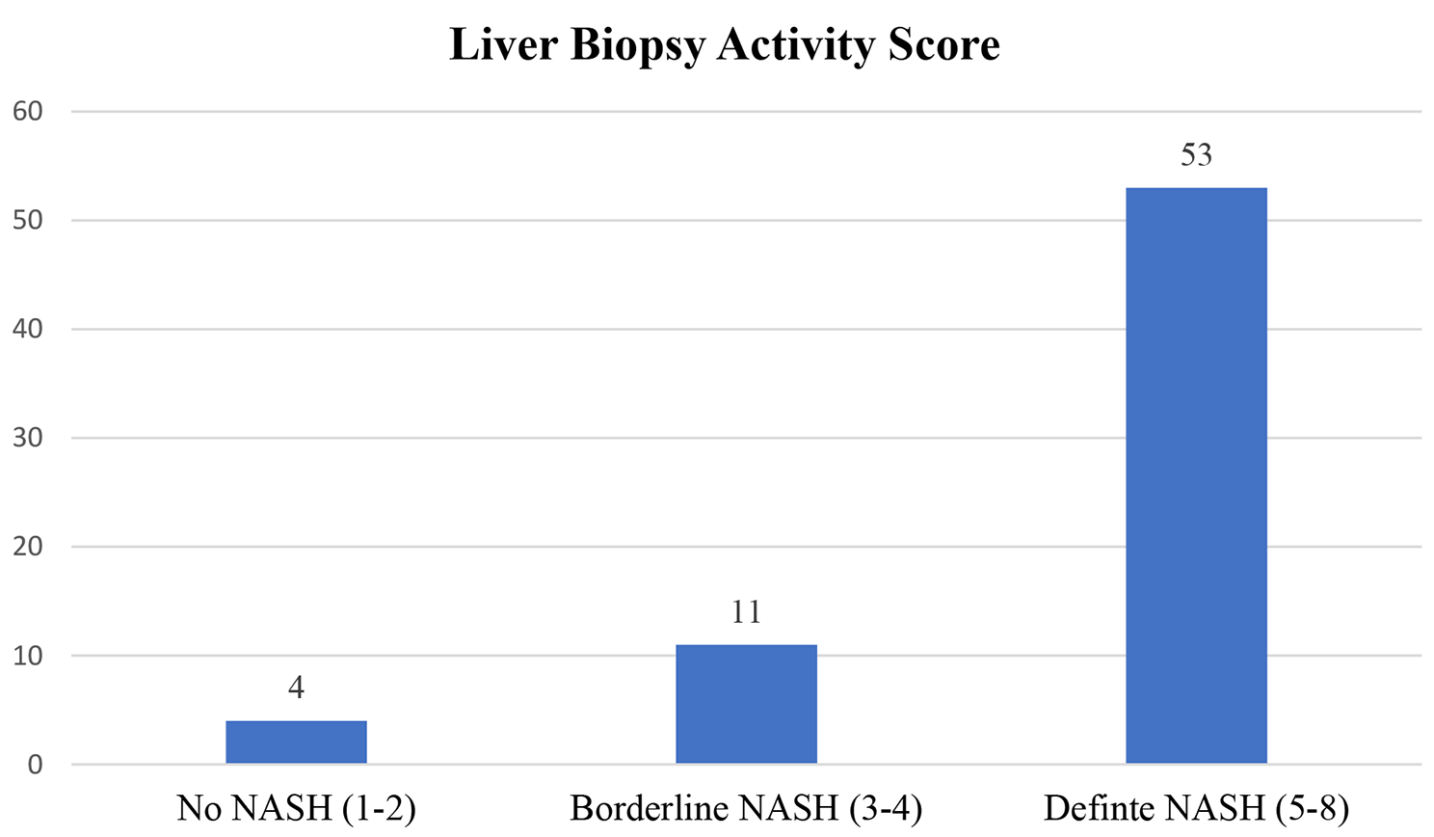
Frequency of NASH-CRN scores among the study participants

Using the NASH CRN score for liver biopsy grading, 53 participants (77.9%) with a CRN score of 5–8 had definite NASH, 11 participants (16.2%) with a CRN score of 3–4 had borderline NASH, and 4 participants (5.9%) with a score of 1–2 did not have NASH (Fig. 2).

According to the ALT values, 51 patients had normal ALT levels (< 40 IU/L); among these patients, 39 (76.5%) had NASH, and 12 (23.5%) did not. Similarly, among the 17 patients with MAFLD with elevated ALT levels (> 40 IU/L), 14 (82.4%) had NASH, while 3 (17.6%) did not (Table 4).

**Table 4:** ALT values and steatohepatitis incidence status

Variables	NASH CRN SCORE CLASSIFICATION	
	No NASH *N* = 15	NASH *N* = 53
**Serum ALT Level**		
Normal (less than 40 IU/L) *N* = 51	12 (23.5%)	39 (76.5%)
Elevated (40 IU/L and above) *N* = 17	3 (17.6%)	14 (82.4%)

X^2^ = 0.257, *P* = 0.446. Fisher’s = 0.745, *P* = 0.446. **No NASH** – Liver biopsy activity score of 1–4

**NASH**- Liver biopsy activity score of 5 or above. **ALT**- alanine transferase

Patients with higher grades of fatty liver disease on abdominal ultrasound scan also had a greater incidence of NASH; 61.9% of patients with grade 1 steatosis on ultrasound had NASH; 84.1% of patients with grade 2 steatosis on ultrasound had NASH; and 100% of patients with grade 3 steatosis on ultrasound had NASH. Additionally, patients with definite NASH according to histology had higher proportions of Grade 1, 2 and 3 fatty liver disease according to ultrasound (61.9%, 84.1% and 100%, respectively), while the proportions of patients with steatosis and without NASH according to ultrasound were as follows: Grade 1 (38.1%), Grade 2 (15.9%) and Grade 3 (0%). There was a weak correlation between the grade of fatty liver disease determined by ultrasound and NASH incidence (*p* = 0.027) (Tables 5 & 6).

**Table 5:** Relationships between ultrasound grade and NASH grade

Variables	NASH Classification	
	No NASH^*^ n (%)	Definite NASH ^**^ n (%)
Ultrasound Grading		
Grade 1 Grade 2 Grade 3	8 (38.1) 7 (15.9) 0 (0)	13 (61.9) 37 (84.1) 3 (100.0)

Likelihood ratio- 5.292, *p* = 0.071; Pearson correlation coefficient = 0.27 (*p* = 0.027)

*No NASH indicates a NASH CRN score of 1–4

**NASH indicates a NASH CRN score of 5 or above

**Table 6:** Associations between serum ALT levels and USS grade and hepatic histological findings

	Variables	Serum ALT level		Statistics	*P* value
		Normal *n* (%) = 51	Elevated *n* (%) = 17		
Ultrasound Grading	Grade 1 Grade 2 Grade 3	15 (71.4) 34 (77.3) 2 (66.7)	6 (28.6) 10 (22.7) 1 (33.0)	LR = 0.367	*p* = 0.832
Histology steatosis grading (S)	Grade 1 (5–33%) Grade 2 (34–66%) Grade 3 (> 66%)	14 (82.4) 19 (82.6) 18 (64.3%)	3 (17.6) 4 (17.4) 10 (35.7%	LR = 2.882	*P* = 0.237
Hepatocyte ballooning (B)	Few Many No	24 (77.4) 20 (66.7) 7 (100)	7 (22.6) 10 (33.3) 0 (0)	X^2^ = 3.541	*P* = 0.170
Lobular inflammation (L)	Yes No	47 (73.4) 4 (100.0)	17 (26.6) 0 (0.0)	Fisher’s = 1.827	*p* = 0.565
Stages of fibrosis	Stage 0 Stage 1 Stage 2 Stage 3	11 (73.3) 20 (76.9) 19 (79.2) 1 (33.3)	4 (26.7) 6 (23.1) 5 (20.8) 2 (66.7)	LR = 2.607	*p* = 0.456
Lobular lipogranulomas	Yes No	15 (68.2) 36 (78.3)	7 (31.8) 10 (21.7)	X^2^ = 0.806	*p* = 0.369
Mallory Denk Bodies	Yes No	9 (75) 42 (75)	3 (25) 14 (25)	X^2^ = 0.000	*P* = 1.000

**Stage 0-** no fibrosis, **stage 1-** perisinusoidal or periportal fibrosis, **stage 2-** portal and perisinusoidal fibrosis, **stage 3-** bridging fibrosis, **stage 4-** cirrhosis

**Fig. 3:**
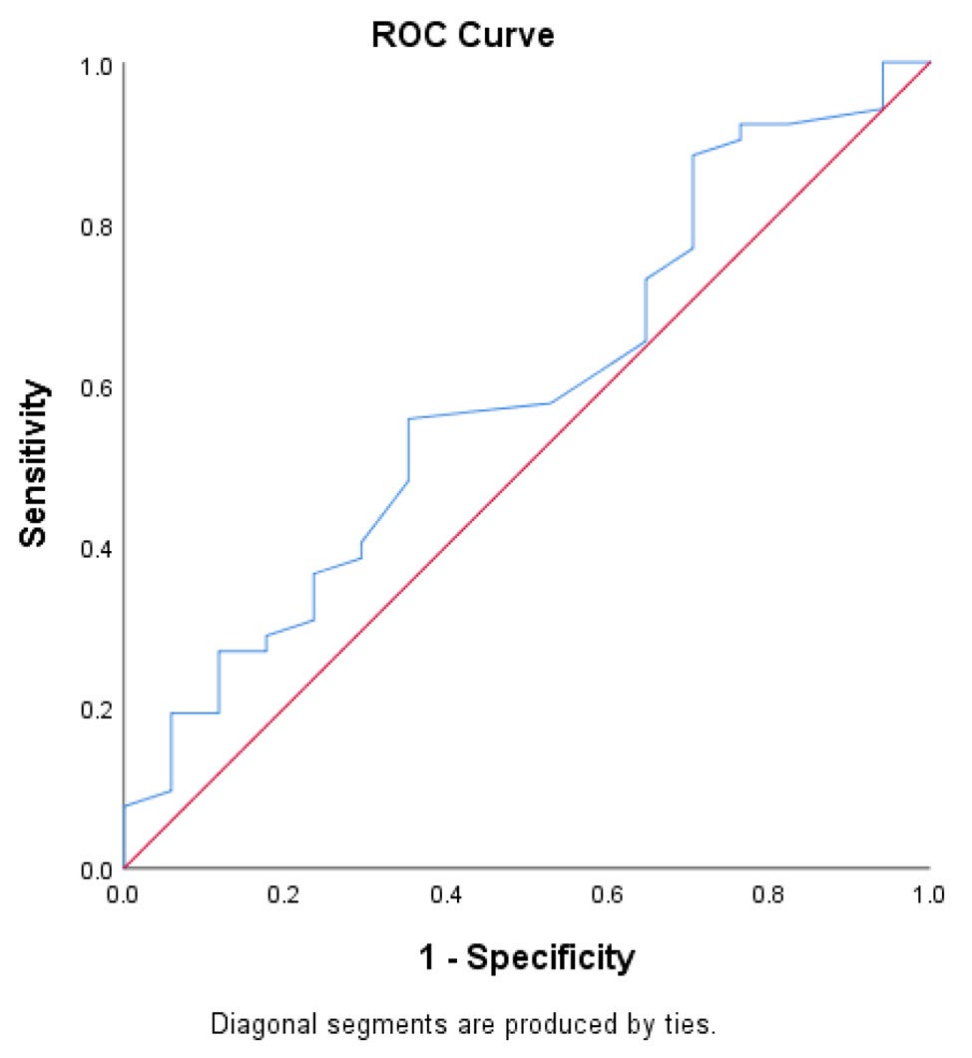
ROC curves for the NASH CRN score and serum ALT concentration

The ROC curves of the NASH CRN score and serum ALT concentration showed that the area under the curve (diagnostic accuracy) was 0.590 (*p* = 0.268, 95% CI = 0.435–0.745). At an ALT cut-off value of 27.5 IU/L, the sensitivity and specificity were 55.8% and 64.7%, respectively, with positive and negative predictive values of 82.9% and 32.4% (Fig. 3; Table 7).

**Table 7:** ROC parameters

ROC Parameters	Value
Area under the curve (Diagnostic accuracy)	0.590 (*p*-value = 0.268; 95% CI = 0.435–0.745)
Cut off value	27.5
Sensitivity	55.8%
Specificity	64.7%
Positive predictive value	82.9%
Negative predictive value	32.4%

## Discussion

The majority of the patients with MAFLD were females, with a female-to-male ratio of 1.73:1. Studies including premenopausal women have reported male-dominated findings, while in studies including postmenopausal women, the effect of hormonal protection was lost; therefore, sex differences may be the same, or female MAFLD patients may dominate [1]. Most women in this study had reached menopause, with a mean age of 49.93 *±* 12 years, which may explain why females predominated in this study. Additionally, there was no significant difference between the mean age of the participants with normal and elevated ALT levels or between those with and without NASH. These findings are consistent with the findings of Uslusoy et al. [14].

This study revealed that the overall risk factors for MAFLD were hypertension, type 2 DM, dyslipidaemia, obesity, and impaired fasting glucose. Moreover, more patients with NASH had these conditions than did those without NASH; these findings are similar to previous reports [15–18].

The most widely used imaging test for hepatic steatosis is ultrasound because it is readily available, inexpensive, and safe [2]. The majority of the patients in this study had grade 2 hepatic steatosis on ultrasound. This study revealed that the greater the grade of steatosis on ultrasound was, the greater the proportion of participants with definite NASH. There is a paucity of data in the literature concerning the ability of ultrasound to distinguish between simple steatosis and histological features of NASH. Saadeh et al. [19] previously reported that none of the radiological modalities could detect hepatocyte features important for diagnosing NASH. This study further corroborated the findings of Saadeh et al. [19] However, there was no statistically significant relationship between USS for hepatic steatosis and NASH incidence. A statistically weak relationship was observed between the grade of hepatic steatosis determined by ultrasound and the incidence of NASH. Similar findings were reported by Abdel et al. [20] Therefore, this study showed that there was a significant correlation between the USS grade and the presence of NASH and the NASH CRN score.

ALT levels were measured using a cut-off value of 40 IU/L, which is the standard laboratory reference value based on the kit that was used in the automated Cobas machine and is comparable to the International Federation of Clinical Chemistry Method. Three quarters (75%) of the participants had normal ALT levels (< 40 IU/L), while one quarter (25%) had elevated ALT levels (> 40 IU/L). Ma et al. [21] reported that only 25% of the patients had normal ALT levels in a systematic review and meta-analysis. This study showed that the ALT level does not reflect what occurs in the liver of a patient with MAFLD, which is consistent with earlier reports [21–23]. These findings may be explained by the findings of Ma et al. [21], who showed that a normal ALT level is closely associated with diabetes, metabolic syndrome and steatosis grade 1 compared with steatosis grades 2 and 3. It was also associated with sex, and females with NAFLD tended to have normal ALT levels.

This study also showed that more patients with normal ALT levels had simple steatosis than did those with elevated ALT levels. Wong et al. [18] and Khosravi et al. [24] found that ALT levels did not correlate with histological parameters in patients with MAFLD. Wong et al. [15] reported that NASH and significant fibrosis can be found in participants with normal ALT levels. Another multicentre study involving 733 patients with MAFLD who underwent liver biopsy reported that advanced fibrosis did not correlate with alanine transaminase (ALT) levels [25]. This study revealed variability in ALT levels and the incidence of NASH using the NASH CRN score. The prevalence of NASH did not differ between patients with normal ALT levels and those with elevated ALT levels. Similarly, other authors have reported varying percentages of patients with NASH and normal ALT levels and MAFLD [26–28].

This study revealed significant differences in BMI, systolic blood pressure and AST levels between patients with and without NASH and between patients with and without elevated ALT levels. These findings suggested that patients with MAFLD who are obese and hypertensive with elevated ALT and AST levels are at increased risk of progression to NASH.

This study revealed that the frequency of biopsy-confirmed NASH was highly comparable to that of another report by Ajmera et al. [29] A meta-analysis by Dufour et al. [30] reported a wide range of (15.9–68.3%) of patients with MAFLD who underwent liver biopsy and were confirmed to have NASH.

There is no specific cut-off value for ALT in patients with MAFLD. The area under the receiver operating curve (AUROC) for ALT levels relating to the incidence of NASH was 0.590. At an ALT cut-off value of 27.5 IU/L, the sensitivity and specificity were 55.8% and 64.7%, respectively. The study failed to demonstrate an optimal ALT level that would mostly predict the incidence of NASH. These findings are comparable with those of other studies of patients with MAFLD [18, 28].

### Limitations of the study

The intake of alcohol was estimated by patient recall, which may not be accurate.

The sample size of the study was small, and a larger sample size would have increased the power of the study.

This was a cross-sectional study, and patients were not followed up to determine what would have become of those without NASH; it is possible that some may have progressed to NASH.

The ultrasonography technique used to detect hepatic fat in this study had poor sensitivity for diagnosing steatosis when it was less than 30%. A more advanced test, such as magnetic resonance imaging (MRI), including magnetic resonance spectroscopy, would have been a better option because it can detect the presence of hepatic fat greater than 5.6%, which is the defining threshold with an accuracy of 100%.

### Strength of the study

Liver biopsy and histology were performed for all patients with MAFLD irrespective of their serum ALT levels. This approach is the gold standard for diagnosing NASH and detecting early fibrosis, cirrhosis, and hepatocellular carcinoma.

## Conclusion

The proportion of patients with NASH and normal ALT levels was greater than that of patients with NASH and elevated ALT levels. The variability in ALT levels and the histological diagnosis of NASH showed that the ALT level is not a reliable marker for diagnosing NASH. In addition, the sensitivity and specificity of the ALT level for predicting NASH were found to be low. There is no ideal cut-off value for ALT levels using the AUROC for predicting the incidence of NASH. Thus, liver biopsy may be necessary for all patients with MAFLD, irrespective of their ALT level, to detect the presence of NASH.

This study also showed that despite the increasing incidence of NASH with increasing steatosis grade on ultrasound, there was a weak correlation between the grade of steatosis on ultrasound and the incidence of NASH.

## Data Availability

The datasets used and/or analysed during the current study available from the corresponding author on reasonable request.

## Abbreviations

ALT: Alanine Aminotransferase
AST: Aspartate Aminotransferase
AUROC: Area under receivers operating curve
BMI: Body Mass Index
CRN: Clinical Research Network
DM: Diabetes mellitus
T2DM: Type 2 Diabetes mellitus
HDL-C: High density lipoprotein cholesterol
HIV: Human Immunodeficiency Virus
LDL-C: Low density lipoprotein cholesterol
LFT: Liver function test
MRI: Magnetic Resonance Imaging
MetS: Metabolic syndrome
MAFLD: Metabolic associated Fatty Liver Disease
NAFLD: Non-alcoholic fatty liver disease
NASH: Non-alcoholic Steatohepatitis
NHANES: National Health and Nutrition Examination Survey
NHREC: National Health Research Ethics committee
OAUTHC: Obafemi Awolowo University Teaching Hospital Complex
ROC: Receiver operating curve
SD: Standard deviation
SPSS: Statistical package for the social sciences
TC: Total Cholesterol
USA: United States of America
USG: Ultrasonography
USS: Ultrasound scan
WHO: World Health Organisation
